# Assessment of BeiDou-3 and Multi-GNSS Precise Point Positioning Performance

**DOI:** 10.3390/s19112496

**Published:** 2019-05-31

**Authors:** Guoqiang Jiao, Shuli Song, Yulong Ge, Ke Su, Yangyang Liu

**Affiliations:** 1 Shanghai Astronomical Observatory, Chinese Academy of Sciences, Shanghai 200030, China; jiaoguoqiang@shao.ac.cn (G.J.); ksu@shao.ac.cn (K.S.); 2 University of Chinese Academy of Sciences, Beijing 100049, China; geyulong15@mails.ucas.ac.cn (Y.G.); liuyangyang17@mails.ucas.ac.cn (Y.L.); 3 National Time Service Center, Chinese Academy of Sciences, Xi’an 710600, China

**Keywords:** BeiDou-3, international GNSS Monitoring and Assessment System (iGMAS), PDOP, precise point positioning (PPP), positioning accuracy, convergence time, ZTD

## Abstract

With the launch of BDS-3 and Galileo new satellites, the BeiDou navigation satellite system (BDS) has developed from the regional to global system, and the Galileo constellation will consist of 26 satellites in space. Thus, BDS, GPS, GLONASS, and Galileo all have the capability of global positioning services. It is meaningful to evaluate the ability of global precise point positioning (PPP) of the GPS, BDS, GLONASS, and Galileo. This paper mainly contributes to the assessment of BDS-2, BDS-2/BDS-3, GPS, GLONASS, and Galileo PPP with the observations that were provided by the international Global Navigation Satellite System (GNSS) Monitoring and Assessment System (iGMAS). The Position Dilution of Precision (PDOP) value was utilized to research the global coverage of GPS, BDS-2, BDS-2/BDS-3, GLONASS, and Galileo. In particular, GPS-only, BDS-2-only, BDS-2/BDS-3, GLONASS-only, Galileo-only, and multi-GNSS combined PPP solutions were analyzed to verify the capacity of the PPP performances in terms of positioning accuracy, convergence time, and zenith troposphere delay (ZTD) accuracy. In view of PDOP, the current BDS and Galileo are capable of global coverage. The BDS-2/BDS-3 and Galileo PDOP values are fairly evenly distributed around the world similar to GPS and GLONASS. The root mean square (RMS) of positioning errors for static BDS-2/BDS-3 PPP and Galileo-only PPP are 10.7, 19.5, 20.4 mm, and 6.9, 18.6, 19.6 mm, respectively, in the geographic area of the selected station, which is the same level as GPS and GLONASS. It is worth mentioning that, by adding BDS-3 observations, the positioning accuracy of static BDS PPP is improved by 17.05%, 24.42%, and 35.65%, and the convergence time is reduced by 27.15%, 27.87%, and 35.76% in three coordinate components, respectively. Similar to the static positioning, GPS, BDS-2/BDS-3, GLONASS, and Galileo have the basically same kinematic positioning accuracy. Multi-GNSS PPP significantly improves the positioning performances in both static and kinematic positioning. In terms of ZTD accuracy, the difference between GPS, BDS-2/BDS-3, GLONASS, and Galileo is less than 1 mm, and the BDS-2/BDS-3 improves ZTD accuracy by 20.48% over the BDS-2. The assessment of GPS, BDS-2, BDS-2/BDS-3, GLONASS, Galileo, and multi-GNSS global PPP performance are shown to make comments for the development of multi-GNSS integration, global precise positioning, and the construction of iGMAS.

## 1. Introduction

In addition to GPS and GLONASS equipped with the global positioning capability, both the BeiDou navigation satellite system (BDS) and Galileo have global positioning capability with the launch of the BDS-3 and Galileo satellites. Especially, BDS has developed from a regional to global system with the open service of the global basic navigation service of BDS-3 in December 2018 and the Galileo constellation has reached 26 satellites in space. The construction of the BDS follows the “three-step” strategy, including the demonstration system BDS-1, the regional system BDS-2, and the global system BDS-3 [1]. BDS-1 contains three Geostationary Earth Orbit (GEO) satellites, the first of which was launched in 2000 and the entire BDS-1 system was completed in 2003 [2]. The master control station using the Radio Determination Satellite System (RDSS) of BDS-1 [3] can determine the user location. BDS-2 contains five GEO satellites, seven Inclined Geosynchronous Satellite Orbit (IGSO) satellites, and four Medium Earth Orbit (MEO) satellites, which can provide positioning and navigation services for users over the whole Asian-Pacific region by the end of 2012 [4]. The construction of BDS-3 began in 2015. BDS-3 contains five GEO, 27 MEO, and three IGSO, and it will provide positioning and navigation services to global users by 2020 [5]. The European Galileo is the third Global Navigation Satellite System (GNSS), which can offer a continuous, flexible, and precise positioning service. When compared with BDS, the development of the Galileo follows the two steps. Four In-Orbit Validation (IOV) satellites have been successfully launched and they are in orbit. Currently, the IOV phase is closed and it is a transition phase to Full Operational Capability (FOC) [6]. The Galileo constellation has reached 26 satellites in space, completing the constellation in July 2018.

In addition that, GPS and GLONASS perform global precise point positioning (PPP) calculations, BDS and Galileo also have this ability already with the launch of BDS-3 and Galileo new satellites. The assessment of the precise positioning ability of the GPS, BDS-2, BDS-2/BDS-3, GLONASS, Galileo, and multi-GNSS has become very meaningful in GNSS development. Especially, the technology of BDS-3 and GNSS combined PPP has become very important in the development of multi-GNSS integration. In the terms of the assessment of the precise positioning ability of the different navigation system, many scholars have made some achievements. Xia et al. indicated that the contribution of Galileo can improve the positioning accuracy by 23.7%~29.96% and 10.59%~11.07% and can reduce the convergence time by 45.48% and 11.04% for GPS-only and GPS/GLONASS kinematic PPP, respectively [7]. Xu et al. concluded that integrating the BeiDou experimental satellites (BDS-3e) could slightly improve the horizontal precision for BDS-2-only or GPS/BDS-2 combined solutions by comparing GPS/BDS-2, GPS/BDS-2/BDS-3, BDS-2, and BDS-3 PPP [8]. The addition of BDS-2, Galileo and GLONASS systems to the standard GPS-only PPP can reduce the convergence time and improve the positioning accuracy, and the position series of multi-PPP are much more stable than the GPS-only solutions [9,10]. The previous research on the assessment of GNSS performance based on multi-GNSS PPP mainly focused on BDS-2 and its benefit to other navigation systems. Although the relative positioning and PPP technology were used to conclude that the BDS-3e could contribute to improving performance of precise positioning [2,11], there are few researches regarding the assessment of BDS-2/BDS-3 PPP and its contribution to multi-GNSS based on the raw BDS-3 observation. In addition, the previous discussion on the assessment of GNSS performance mainly focused on the positioning accuracy and convergence time, with less consideration for zenith troposphere delay (ZTD) accuracy. For the above reasons, this paper conducts GPS, BDS-2, BDS-2/BDS-3, GLONASS, Galileo, and multi-GNSS PPP tests and evaluates the performance of each system in term of positioning accuracy, convergence time, and ZTD accuracy based on international GNSS Monitoring and Assessment System (iGMAS) observation data and the precise products that were provided by Wuhan University (WHU). This paper makes comments for the development of multi-GNSS integration, global precise positioning, and the construction of iGMAS by evaluating the performance of GPS, BDS-2, BDS-2/BDS-3, GLONASS, Galileo, and multi-GNSS global PPP.

The remaining paper is organized, as follows: PPP methods are first presented in Section 2. Experiment and data processing strategies are then described in Section 3. In Section 4, the Position Dilution of Precision (PDOP) was used to explore the service areas of the GPS, BDS-2, BDS-2/BDS-3, GLONASS, Galileo, and multi-GNSS and compare their differences. In Section 5, The GPS, BDS-2, BDS-2/BDS-3, GLONASS, Galileo PPP, and multi-GNSS PPP are used to verify the capacity of the global precise positioning in terms of positioning accuracy, convergence time, and ZTD accuracy. In the last section, the conclusions are drawn.

## 2. Multi-GNSS Ionospheric-Free PPP Model

The ionospheric-free (IF) model is generally utilized in PPP to mitigate the first order ionospheric delay with dual-frequency pseudorange and carrier-phase observations and the higher-order ionospheric delay is absorbed by observation noises, which can be expressed as [12,13]

(1) $$\left\{ \begin{array}{l} P_{\mathrm{IF}}=R_{r}^{s}+\left(c\cdot \delta t_{r}+l_{b_{P}}\right)-\left(c\cdot \delta t^{s}+l_{B_{P}}\right)+T_{r}^{s}+\varepsilon_{P}\\ \Phi_{\mathrm{IF}}=R_{r}^{s}+\left(c\cdot \delta t_{r}+l_{b_{\phi }}\right)-\left(c\cdot \delta t^{s}+l_{B_{\phi }}\right)+T_{r}^{s}+\alpha_{1,2}\cdot \lambda_{1}\cdot N_{1}+\beta_{1,2}\cdot \lambda_{2}\cdot N_{2}+\varepsilon_{\Phi } \end{array}\right.$$

with

(2) $$\left\{ \begin{array}{l} \alpha_{1,2}=\frac{f_{1}^{2}}{f_{1}^{2}-f_{2}^{2}},\;\beta_{1,2}=\frac{-f_{2}^{2}}{f_{1}^{2}-f_{2}^{2}}\\ l_{B_{P}}=\alpha_{1,2}\cdot B_{P_{1}}+\beta_{1,2}\cdot B_{P_{2}},l_{b_{P}}=\alpha_{1,2}\cdot b_{P_{1}}+\beta_{1,2}\cdot b_{P_{2}}\\ l_{B_{\phi }}=\alpha_{1,2}\cdot B_{\phi_{1}}+\beta_{1,2}\cdot B_{\phi_{2}},l_{b_{\phi }}=\alpha_{1,2}\cdot b_{\phi_{1}}+\beta_{1,2}\cdot b_{\phi_{2}} \end{array}\right.$$

where $P_{\mathrm{IF}}$ is pseudorange IF observation; $\Phi_{\mathrm{IF}}$ is the IF carrier-phase observation; $R_{r}^{s}$ denotes the computed geometrical range; $T_{r}^{s}$ is the tropospheric delay;$B_{P_{i}}$ and $b_{P_{i}}$ are the frequency-dependent satellite and receiver uncalibrated code delays (UCDs); $B_{\phi_{i}}$ and $b_{\phi_{i}}$ are the frequency-dependent satellite and receiver uncalibrated phase delays (UPDs); N_1_ and N_2_ are the integer phase ambiguity on the frequency $f_{1}$ and $f_{2}$; $\lambda_{1}$ and $\lambda_{2}$ are the wavelengths of the different carrier phase; $\varepsilon_{P}$ and $\varepsilon_{\Phi }$ are the pseudorange and carrier phase observation noises and multipath error for pseudorange and carrier observation; and, $\alpha_{1,2}$ and $\beta_{1,2}$ are the frequency-dependent factors, which are independent of the satellite number.

By convention, the IGS precise satellite clock products are generated using IF observables and the satellite clock offsets absorb the IF combination of satellite UCDs [14], which can be expressed, as follows

(3) $$c\cdot \delta t_{\mathrm{IF}}^{s}=c\cdot \delta t^{s}+\alpha_{1,2}\cdot B_{P_{1}}+\beta_{1,2}\cdot B_{P_{2}}$$

The influence of the satellite UCDs can be eliminated if we use the observations of the same frequency as the satellite clock product for PPP. Furthermore, we use the observation whose frequency is different from satellite clock offset, the differential code bias (DCB) products could be used to correct the satellite UCDs [12,15], which can be expressed as

(4) $$\left\{ \begin{array}{l} P_{\mathrm{IF}}=R_{r}^{s}+\left(c\cdot \delta t_{r}+l_{b_{P}}\right)-c\cdot \delta t_{\mathrm{IF}}^{s}+T_{r}^{s}+\varepsilon_{P}\\ \Phi_{\mathrm{IF}}=R_{r}^{s}+\left(c\cdot \delta t_{r}+l_{b_{P}}\right)-c\cdot \delta t_{\mathrm{IF}}^{s}+T_{r}^{s}+\lambda_{\mathrm{IF}}\cdot N_{\mathrm{IF}}+\varepsilon_{\Phi } \end{array}\right.$$

with

(5) $$\lambda_{\mathrm{IF}}\cdot N_{\mathrm{IF}}=\left(l_{b_{\phi }}-l_{B_{\phi }}\right)-\left(l_{b_{P}}-l_{B_{P}}\right)+\alpha_{1,2}\cdot \lambda_{1}\cdot N_{1}+\beta_{1,2}\cdot \lambda_{2}\cdot N_{2}$$

where $N_{\mathrm{IF}}$ is the float ambiguity.

In order to investigate the ability of the combined PPP global positioning, the equations of multi-GNSS PPP can be established, as follows:

(6) $$\left\{ \begin{array}{l} P_{\mathrm{IF}}^{{\mathrm{SYS}}_{1}}=R_{r}^{s}+\left(c\cdot \delta t_{r}+l_{b_{P}}^{{\mathrm{SYS}}_{1}}\right)-c\cdot \delta t_{\mathrm{IF}}^{s}+T_{r}^{s}+\varepsilon_{P}\\ \vdots \\ P_{\mathrm{IF}}^{{\mathrm{SYS}}_{i}}=R_{r}^{s}+\left(c\cdot \delta t_{r}+l_{b_{P}}^{{\mathrm{SYS}}_{1}}\right)-c\cdot \delta t_{\mathrm{IF}}^{s}+{\mathrm{ISB}}_{r}^{{\mathrm{SYS}}_{i}-{\mathrm{SYS}}_{1}}+T_{r}^{s}+\varepsilon_{P}\\ \Phi_{\mathrm{IF}}^{{\mathrm{SYS}}_{1}}=R_{r}^{s}+\left(c\cdot \delta t_{r}+l_{b_{P}}^{{\mathrm{SYS}}_{1}}\right)-c\cdot \delta t_{\mathrm{IF}}^{s}+T_{r}^{s}+\lambda_{\mathrm{IF}}^{{\mathrm{SYS}}_{1}}\cdot N_{\mathrm{IF}}^{{\mathrm{SYS}}_{1}}+\varepsilon_{\Phi }\\ \vdots \\ \Phi_{\mathrm{IF}}^{{\mathrm{SYS}}_{i}}=R_{r}^{s}+\left(c\cdot \delta t_{r}+l_{b_{P}}^{{\mathrm{SYS}}_{1}}\right)-c\cdot \delta t_{\mathrm{IF}}^{s}+{\mathrm{ISB}}_{r}^{{\mathrm{SYS}}_{i}-{\mathrm{SYS}}_{1}}+T_{r}^{s}+\lambda_{\mathrm{IF}}^{{\mathrm{SYS}}_{i}}\cdot N_{\mathrm{IF}}^{{\mathrm{SYS}}_{1}}+\varepsilon_{\Phi } \end{array}\right.$$

with

(7) $${\mathrm{ISB}}_{r}^{{\mathrm{SYS}}_{i}-{\mathrm{SYS}}_{1}}=l_{b_{P}}^{{\mathrm{SYS}}_{i}}-l_{b_{P}}^{{\mathrm{SYS}}_{1}}$$

where ${\mathrm{ISB}}_{r}^{{\mathrm{SYS}}_{i}-{\mathrm{SYS}}_{1}}$ is the inter-system bias (ISB) between the navigation system ${\mathrm{SYS}}_{i}$ and ${\mathrm{SYS}}_{1}$. 

## 3. Data Sets and Processing Strategy

**Figure 1:**
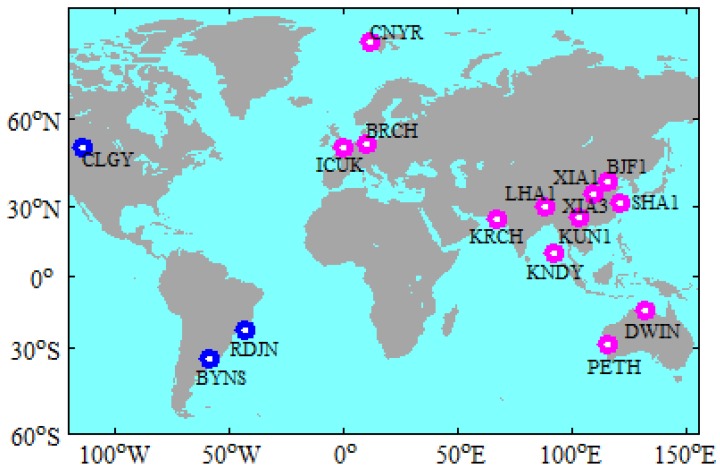
Geographical distribution of the selected stations.

The data were collected by 16 globally distributed iGMAS stations on 30 days, from 2 January 2019 to 31 January 2019. All of the selected stations can receive the observations from GPS, BDS-2, BDS-3, GLONASS, and Galileo constellations [16]. Figure 1 shows the distribution of the selected iGMAS stations. When considering that the BDS-2 is a regional navigation system, the stations in Asia and Europe are selected for BDS-2 PPP to ensure the objectivity of the evaluation. The stations that are marked with red in Figure 1 are used for the BDS-2 PPP. The observation data have a sampling interval of 30 s. The WHU orbit and clock products (ftp://igs.ign.fr/pub/igs/products/mgex/) have a sampling of 15 min. and 30 s, respectively, and the time system of products is GPS time (GPST). The coordinate frame of WHU orbit and clock products is IGS14 [17]. It is worth mentioning that the BDS B1I (1561.098 MHz) and BDS B3I (1268.52 MHz) signals are used to determinate the BDS (BDS-2/BDS-3) satellite orbit and clock offset [18]. The station coordinates in the SINEX file provided by iGMAS (http://112.65.161.230/download/index.php; ftp://222.240.181.170/products/) are used as the references in order to assess the positioning accuracy. It is noteworthy that the difference between the iGMAS and IGS station coordinates precision is in the millimeter level [19,20]. Therefore, iGMAS station coordinates is sufficient to assess the positioning accuracy.

In the IF PPP model, the estimated parameters include the receiver position, the zenith wet tropospheric delay (ZWD), the receiver clock offset, the inter-system bias (ISB), and the ambiguity. These selected iGMAS stations were used for static PPP solutions. The Kalman filtering algorithm is applied in PPP processing. Phase Center Offset (PCO) and Phase Center Variations (PCV) of GPS GLONASS and Galileo satellite and receiver antennas are corrected while using the ANTEX file that was provided by IGS. Regarding the BDS satellite, the ANTEX file that was provided by iGMAS Analysis Center of Shanghai Astronomical Observatory is used to correct the PCO and PCV of BDS. The correction of PCO and PCV of some receiver antennas only gives GPS and GLONASS, and it rarely updates BDS and Galileo [21,22]. We can only use the correction value provided by GPS to correct BDS and Galileo when we make PCO and PCV corrections on the receiver antenna. In addition, the pole coordinates and UTC-UT1 value provided by the WHU ERP products correct the pole tide and the Earth rotation effects. Table 1 summarizes the detailed processing strategy for PPP.

Table 2 shows that the comparison of GPS, BDS-2, BDS-2/BDS-3, GLONASS, and Galileo IF-PPP models, where m, o, n, p, and q are the number of satellites for GPS, BDS-2, BDS-3, GLONASS, and Galileo, respectively. In the figure and table below, G, C2, C, R, E, GC, GR, GE, CR, CE, RE, and GCRE represent GPS, BDS-2, BDS-2/BDS-3, GLONASS, Galileo, GPS/BDS-2/BDS-3, GPS/GLONASS, GPS/Galileo, BDS-2/BDS-3/GLONASS, BDS-2/BDS-3/Galileo, GLONASS/Galileo, and GPS/BDS-2/BDS-3/GLONASS/Galileo, respectively.

## 4. Service Area

In this paper, the Position Dilution of Precision (PDOP) is used to explore the differences between GPS, BDS-2, BDS-2/BDS-3, GLONASS, Galileo, and multi-GNSS in service areas. The global region is divided into grids of 2.5° × 5°. Subsequently, the PDOP values of each grid are calculated using the broadcast ephemeris (brdm0240.19p) on day of year (DOY) 024, 2019. During the computation, the cut-off elevation is set to 7.5° and the time resolution is 10 min. For every grid, one calculation result of the PDOP is obtained at each epoch, which generated a PDOP sequence. Afterwards, we could get the mean value from this sequence for every grid.

**Figure 2:**
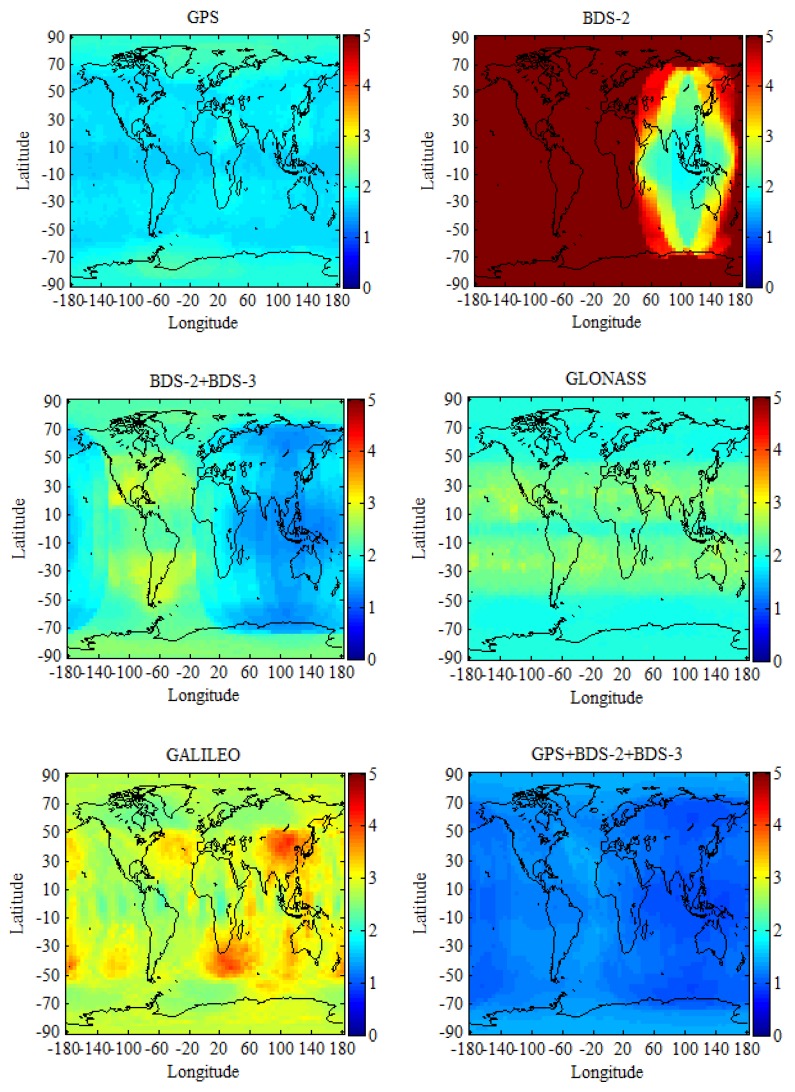

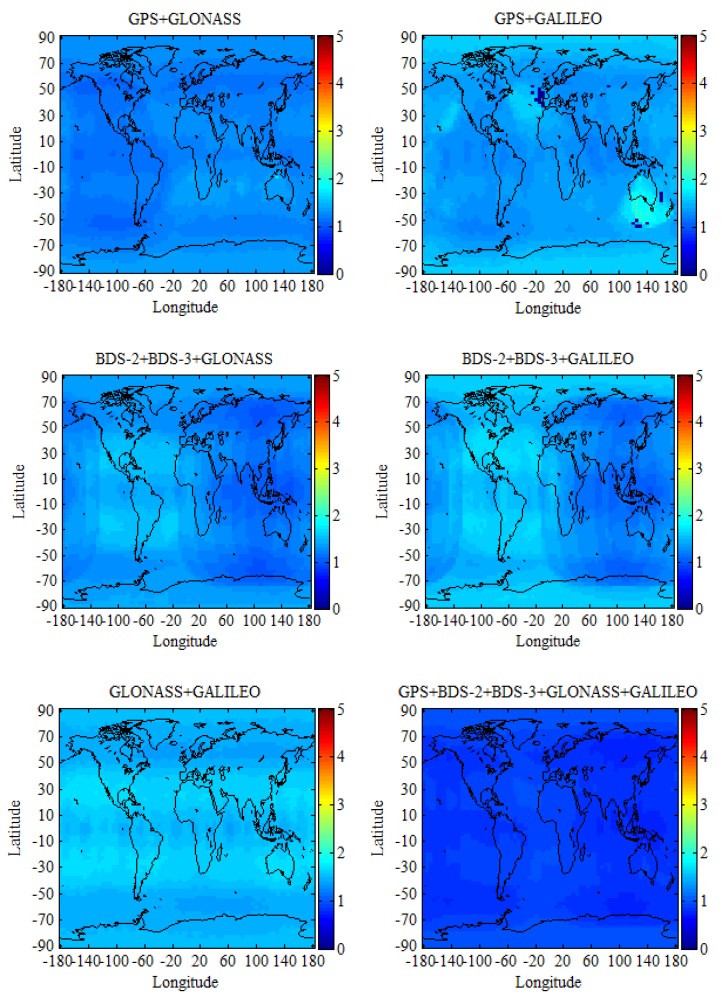
The global mean Position Dilution of Precision (PDOP distribution for GPS, BDS-2, BDS-2/BDS-3, GLONASS, Galileo, and multi-GNSS constellations on DOY 024, 2019, with the elevation cutoff of 7.5°.

Figure 2 shows the global mean PDOP distribution for GPS, BDS-2, BDS-2/BDS-3, GLONASS, Galileo, GPS/GLONASS, GPS/Galileo, GPS/BDS-2/BDS-3, BDS-2/BDS-3/GLONASS, BDS-2/BDS-3/Galileo, GLONASS/Galileo, and GPS/BDS-2/BDS-3/GLONASS/Galileo constellations. It can be seen from Figure 2 (BDS-2) that the BDS-2 service area is mainly centered in the Asia-Pacific region, where its geostationary Earth orbit (GEO) satellites and inclined geosynchronous orbit (IGSO) satellites are mainly concentrated, which results in users in other regions not receiving the signal of BDS-2 or only receiving a small amount of the signal of BDS-2. This is an important reason why BDS-2 does not have global navigation service capability all over the world. When compared BDS-2 and BDS-2/BDS-3 PDOP, the global PDOP of BDS navigation system has significantly decreased with the opening of the global basic navigation service of BDS-3. BDS has full navigation service capability all over the word and it has been transformed from a regional to global navigation system. The performance of BDS will be better when BDS-3 completes the constellation [29]. When compared with GPS, BDS-2/BDS-3, and GLONASS global PDOP, they have basically the same global service performance, except individual regions of the world. From the global PDOP distribution of the Galileo, Galileo has full navigation service capability all over the word, the as same as BDS-2/BDS-3, GPS, and GLONASS. Although the global coverage of each navigation system is already excellent, the problems of the low satellite numbers and the poor satellite constellation structure occur in some special regions of the world when using a single navigation system for positioning and the multi-GNSS is a good way to solve that. The variation ranges of the global mean PDOP of GPS/GLONASS, GPS/Galileo, GPS/BDS-2/BDS-3, BDS-2/BDS-3/GLONASS, BDS-2/BDS-3/Galileo, GLONASS/Galileo, and GPS/BDS-2/BDS-3/GLONASS/Galileo are below 1.5.

## 5. Data Tests and Results Analysis

This section performs GPS-only, BDS-2-only, BDS-2/BDS-3, GLONASS-only, Galileo-only, and multi-GNSS combined PPP tests in order to assess the global performance of GPS, BDS, GLONASS, Galileo, and its contribution to multi-GNSS. The capacity of the multi-GNSS PPP is also verified in terms of its positioning accuracy, convergence time, and ZTD accuracy.

### 5.1. Positioning Accuracy and Convergence Time

#### 5.1.1. Static Results and Analysis

**Figure 3:**
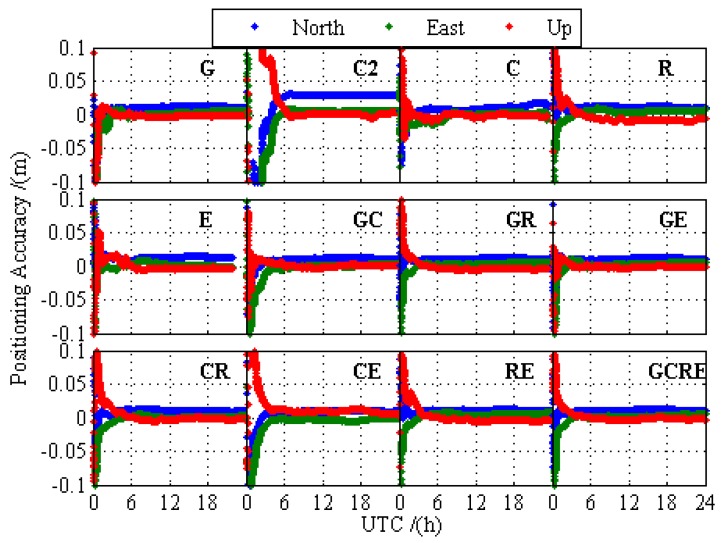
Static PPP positioning errors at BRCH for 12 different processing cases on DOY 024 in 2019.

**Figure 4:**
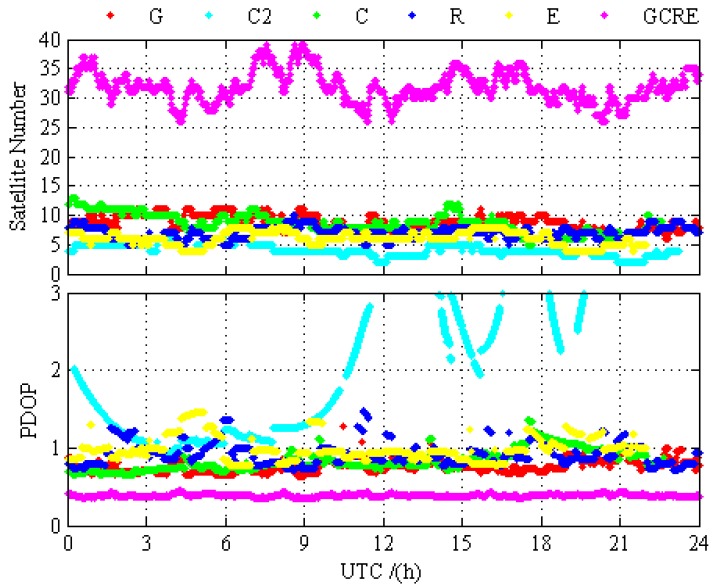
Number of satellites and PDOP at BRCH for GPS-only, BDS-2-only, BDS-2/BDS-3, GLONASS-only, Galileo-only, and GPS/BDS-2/BDS-3/GLONASS/Galileo on DOY 024 in 2019.

In order to compare the difference of the positioning performance between GPS-only, BDS-2-only, BDS-2/BDS-3, GLONASS-only, Galileo-only, GPS/BDS-2/BDS-3, GPS/GLONASS, GPS/Galileo, BDS-2/BDS-3/GLONASS, BDS-2/BDS-3/Galileo, GLONASS/Galileo, and GPS/BDS-2/BDS-3/GLONASS/Galileo, the static PPP processing results for 12 different processing cases are presented and analyzed in this section. Figure 3 and Figure 4 depict the static PPP positioning errors, the number of visual satellite, and PDOP at BRCH for GPS-only, BDS-2-only, BDS-2/BDS-3, GLONASS-only, Galileo-only, and GPS/BDS-2/BDS-3/GLONASS/Galileo, respectively. It is obvious that the problems of the insufficient amount of BDS-2 visual satellite and the poor satellite constellation structure at BRCH are solved with the opening of the global navigation service of BDS-3, and there are no obvious gap between the BDS and other navigation system. When compared with the number of visual satellite and PDOP of single system and multi-GNSS, the measurement fusion technique of multi-GNSS can increase the number of visual satellites and reduce the PDOP value. From the prospect of positioning, no matter GPS, BDS-2, BDS-2/BDS-3, GLONASS, and Galileo, they all have achieved a centimeter-level or even a millimeter-level convergence effect, and multi-GNSS increases the positioning accuracy and the stability of positioning and it decreases the convergence time over the single-constellation and dual-constellation PPP.

**Figure 5:**
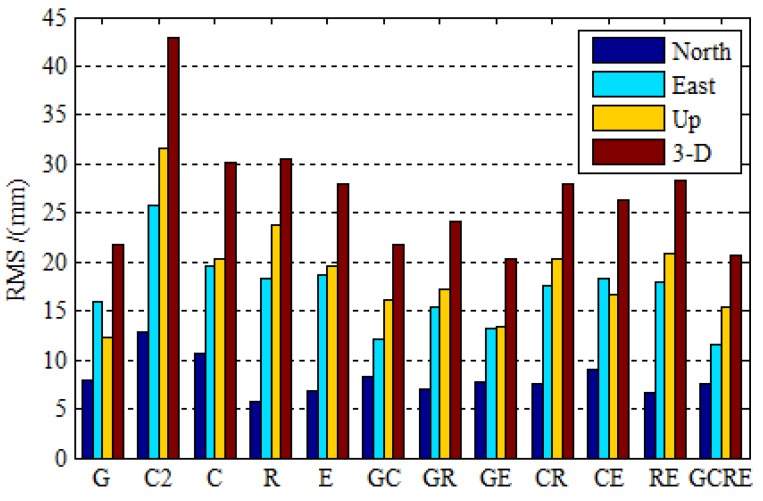
The root mean squares (RMS) error for GPS-only, BDS-2-only, BDS-2/BDS-3, GLONASS-only, Galileo-only, GPS/BDS-2/BDS-3, GPS/GLONASS, GPS/Galileo, BDS-2/BDS-3/GLONASS, BDS-2/BDS-3/Galileo, GLONASS/Galileo, and GPS/BDS-2/BDS-3/GLONASS/Galileo static PPP processing using real observation data collected at 16 stations over 30 days.

Figure 5 depicts the root mean squares (RMS) statistics of 16 stations for 30 days in the north (N), east (E), and up (U) coordinate components, as well as RMS for the three-dimension (3-D) position, in order to scientifically and accurately evaluate the positioning accuracy of each navigation system and its contribution to multi-GNSS. The RMS statistical values using the positioning errors in the last one hour in which the position solutions in all three components have reached stable values. We can draw the following viewpoints based on the solutions of the selected observation data. It is noticed that the BDS-2-only positioning accuracy is comparable to GPS-only and GLONASS-only with a difference. However, there is no obvious disparity between BDS and GPS, GLONASS with the opening of BDS-3. It is worth mentioning that the static BDS-2/BDS-3 PPP improves the position accuracy by 17.05%, 24.42%, and 35.65% over the BDS-2-only PPP in three coordinate components, respectively. The RMS of global position errors for static BDS-2/BDS-3 PPP and Galileo-only PPP are 10.7, 19.5, 20.4 mm, and 6.9, 18.6, 19.6 mm, respectively, as the same level as GPS and GLONASS. However, there is a slight difference of positioning accuracy between BDS, Galileo, and GPS. The PCO and PCV receiver corrections of BDS and Galileo are corrected by using the correction value that was provided by GPS, which could cause the positioning accuracy of BDS and Galileo to be slightly worse than that of GPS. The precise orbit determination strategy of BDS-3 has not yet matured in terms of in-orbit estimations of the phase center correction for the BDS-3 satellites, precise attitude model, and the solar radiation pressure model [30], which is why the positioning accuracy of BDS is slightly worse than that of GPS. Now, turn to the multi-GNSS PPP, the contributions of BDS-2/BDS-3 and Galileo to the improvement of multi-GNSS positioning accuracy. The GPS/BDS-2/BDS-3, GPS/Galileo, BDS-2/BDS-3/GLONASS, BDS-2/BDS-3/Galileo, and GLONASS/Galileo PPP achieves better positioning accuracy than single-constellation PPP. It is worth mentioning that the GPS/BDS-2/BDS-3/GLONASS/Galileo PPP positioning accuracy improvement of 4.75% over the GPS case, 31.45% over BDS-2/BDS-3 case, 32.17% over GLONASS, and 25.81% over Galileo in the three-dimensional (3-D) components is achieved. Additionally, it is clear that the GPS/BDS-2/BDS-3/GLONASS/Galileo PPP positioning accuracy is as good as dual-constellation PPP.

**Figure 6:**
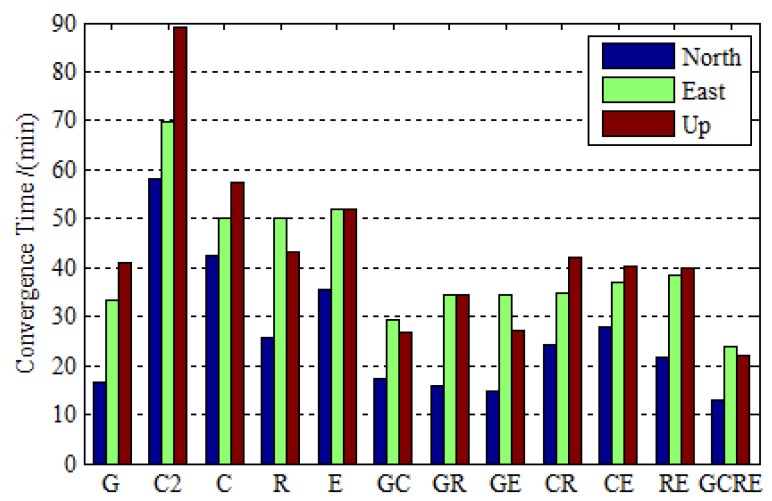
The convergence time for GPS-only, BDS-2-only, BDS-2/BDS-3, GLONASS-only, Galileo-only, GPS/BDS-2/BDS-3, GPS/GLONASS, GPS/Galileo, BDS-2/BDS-3/GLONASS, BDS-2/BDS-3/Galileo, GLONASS/Galileo, and GPS/BDS-2/BDS-3/GLONASS/Galileo static PPP processing using real observation data collected at 16 stations over 30 days.

Figure 6 depicts the convergence time for GPS-only, BDS-2-only, BDS-2/BDS-3 GLONASS-only, Galileo-only, GPS/BDS-2/BDS-3, GPS/GLONASS, GPS/Galileo, BDS-2/BDS-3/GLONASS, BDS-2/BDS-3/Galileo, GLONASS/Galileo, and GPS/BDS-2/BDS-3/GLONASS/Galileo static PPP processing while using real observation data collected at 16 stations over 30 days. In this research, the convergence criterion is defined when the component of positioning errors is less than 0.1 m and keeps within 0.1 m in the subsequent epochs. It is seen that the BDS-2-only PPP takes a much longer time to converge over the GPS-only, GLONASS-only, and Galileo-only PPP. With the development of BDS, the BDS-2/BDS-3 PPP reduces the convergence time by 27.15%, 27.87%, and 35.76% over the BDS-2-only PPP in three coordinate components, respectively. Whilst the BDS performance improves a lot, it is nevertheless a crucial problem that the convergence time is a little poorer than GPS-only PPP. When considering that the precise orbit determination strategy of BDS-3 is still immature and the BDS-3 constellation is not completed, this could be better with the development of the BDS. The performance of Galileo is better in terms of convergence time with the completion of Galileo constellation. Additionally, the convergence time of dual-constellation PPP is reduced over the single-constellation PPP and the GPS/BDS-2/BDS-3/GLONASS/Galileo PPP reduces the convergence time by 20.12%, 27.93%, and 46.09% over GPS-only PPP in the north, east, and up directions, which does further shorten the convergence time over single-constellation and dual-constellation PPP.

#### 5.1.2. Kinematic PPP Results and Analysis

**Figure 7:**
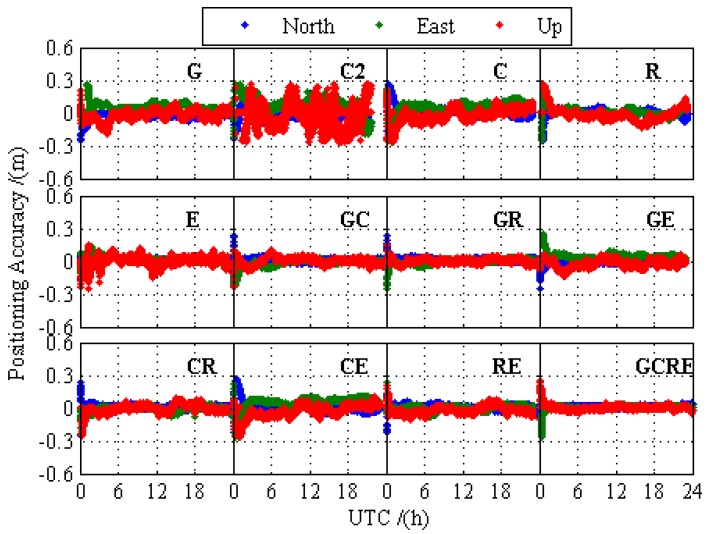
Kinematic PPP positioning errors at KUN1 for 12 different processing cases on DOY 024 in 2019.

**Figure 8:**
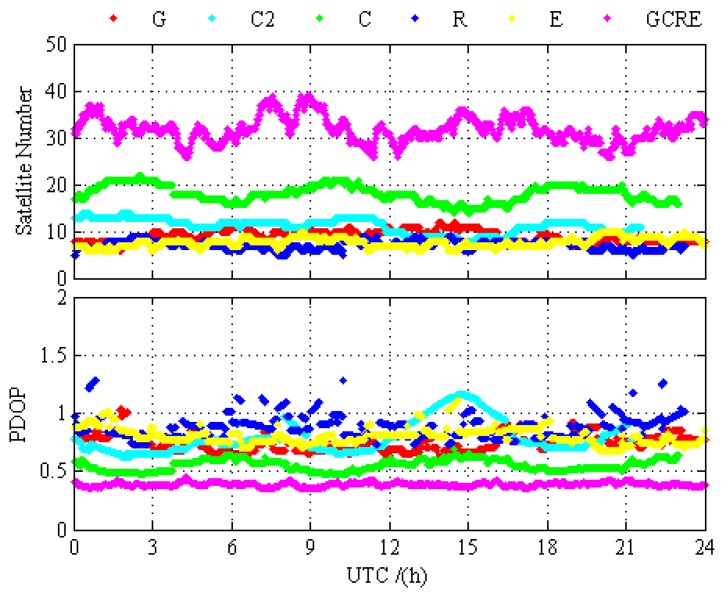
Number of satellite and PDOP at KUN1 for GPS-only, BDS-2-only, BDS-2/BDS-3, GLONASS-only, Galileo-only, and GPS/BDS-2/BDS-3/GLONASS/Galileo on DOY 024 in 2019.

Similar to the static PPP processing, the kinematic PPP processing results for 12 different processing cases are presented and analyzed in this section. The PPP kinematic test was conducted by using real observation data that were collected at 16 stations over 30 days. Figure 7 and Figure 8 show the kinematic PPP positioning errors, the number of visual satellite and PDOP at KUN1 for GPS-only, BDS-2-only, BDS-2/BDS-3, GLONASS-only, Galileo-only, and GPS/BDS-2/BDS-3/GLONASS/Galileo, respectively. Unlike BRCH, the change of the BDS-2-only PDOP of KUN1 is relatively stable, without significant anomaly distortion. As described in Section 4, the BDS-2 service area is mainly centered in the Asia-Pacific region where its GEO satellites and IGSO satellites are mainly concentrated, KUN1 can receive the enough the health signals of BDS-2. There are still obvious gaps between BDS-2 and other navigation system with the enough the health signals of BDS-2 when we compared the kinematic positioning accuracy of BDS-2 and other navigation system. When using the observations of BDS-3, the kinematic positioning accuracy of BDS-2/BDS-3 has been improved obviously compared BDS-2-only kinematic PPP, and there is no obvious noise. The above analyses clearly show that the performance of BDS has been greatly improved with the open service of the global basic navigation service of BDS-3. Similar to static positioning, GPS, BDS-2/BDS-3, GLONASS, and Galileo have all achieved good convergence effect, and multi-GNSS increases the positioning accuracy and the stability of positioning and reduces the convergence time over the single-constellation and dual-constellation PPP.

Table 3 provides the RMS and convergence time in order to assess the performance of the kinematic positioning. The RMS statistical method of kinematic PPP and the convergence criterion are the same as static PPP. We can draw the following viewpoints based on the solutions of the selected observation data. The kinematic BDS-2/BDS-3 PPP improves the position accuracy by 26.32%, 36.00%, and 35.94% and it reduces convergence time by 19.32%, 14.07%, and 19.86% over the BDS-2-only PPP in three coordinate components, respectively. From the perspective of the positioning accuracy and convergence time, there is no significant difference between BDS-2/BDS-3, Galileo, and GPS, GLONASS. BDS and Galileo can improve multi-GNSS positioning accuracy. With the combination of BDS-2/BDS-3 and GPS, the positioning accuracy is improved by 21.59%, 17.47%, and 15.17% and the convergence time is reduced by 35.14%, 8.73%, and 18.49% over the GPS-only case in the three coordinate components, respectively. The GPS/Galileo combined PPP improves the positioning accuracy by 26.36%, 22.10%, and 22.19% and it reduces the convergence time by 32.13%, 24.67%, and 26.19% over the GPS-only PPP in three coordinate components, respectively. Whether positioning accuracy or convergence time, the performance of GPS/BDS-2/BDS-3/GLONASS/Galileo PPP is superior to the single-constellation and dual-constellation PPP. The effect of multi-GNSS to improve the positioning accuracy and reduce convergence time is more obvious in kinematic positioning. When considering that the receiver coordinates are estimated as white noise process, the receiver coordinate parameters cannot be constrained by the explicit kinematics model as in the static processing mode. Therefore, increasing redundant observations can increase the reliability of kinematic solutions.

### 5.2. ZTD Accuracy

When considering that GNSS plays an important role in atmospheric science and ZTD is important to PPP solutions [31,32,33,34]. It is meaningful to assess the performance of the GPS, BDS-2, BDS-2/BDS-3, GLONASS, Galileo, and multi-GNSS PPP in terms of ZTD accuracy. We use conventional methods that ZTD is estimated together with the positioning error parameters in an epoch in order to intuitively illustrate the differences of GPS, BDS-2, BDS-2/BDS-3, GLONASS, Galileo, and multi-GNSS in solving ZTD.

**Figure 9:**
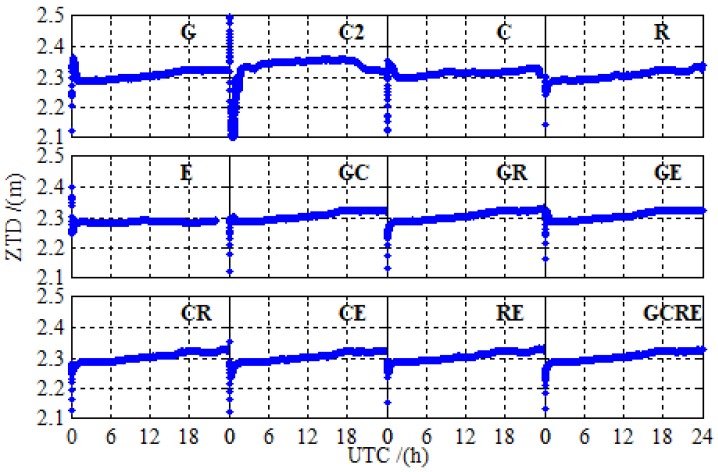
The solutions of zenith troposphere delay (ZTD) at BRCH for 12 different processing cases on DOY 024 in 2019.

Figure 9 shows the solutions of ZTD at BRCH for 12 different processing cases on DOY 024 in 2019. The solutions of ZTD for 12 different processing cases are relatively stable, there are no significant anomaly distortion, and no significant differences between the different systems, except BDS-2. Due to the less of number of BDS-2 available satellites at BRCH, there is a slight difference between the ZTD solutions of BDS-2 and those of other navigation systems. However, adding BDS-3 observations significantly reduces the difference, and the ZTD solutions of BDS-2/BDS-3 are as good as those of other navigation systems.

The ZTD in the tropospheric products provided by iGMAS (http://112.65.161.230/download/; ftp://222.240.181.170/products/) is used to assess the accuracy of ZTD estimated by PPP, since IGS has not yet provided ZTD data for iGMAS stations. In order to verify the accuracy of iGMAS tropospheric products, we counted the RMS of 130 global IGS stations between iGMAS and IGS ZTD products from 2 January 2019 to 31 January 2019. The calculation method of RMS between iGMAS ZTD and IGS ZTD can be expressed as

(8) $$RMS=\sqrt{\frac{\sum_{i=1}^{N}{\left(ZTD_{i}^{iGMAS}-ZTD_{i}^{IGS}\right)}^{2}}{N}}$$

**Figure 10:**
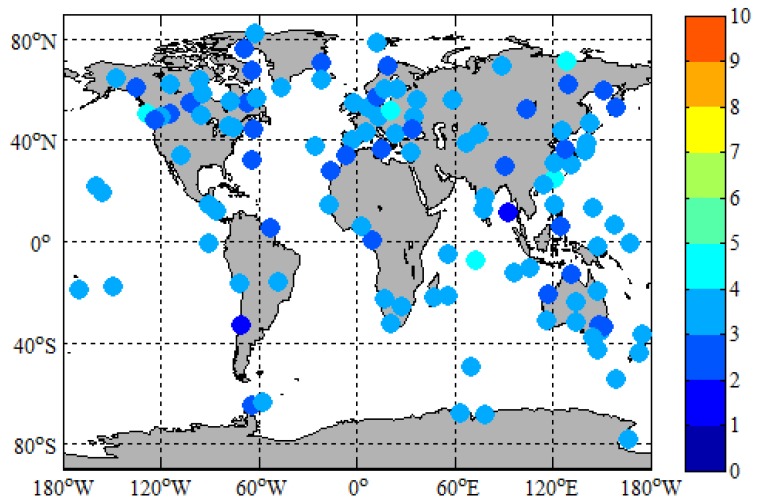
Distribution and 30-day RMS between the international Global Navigation Satellite System Monitoring and Assessment System (iGMAS) and IGS ZTD products of 130 IGS stations.

**Figure 11:**
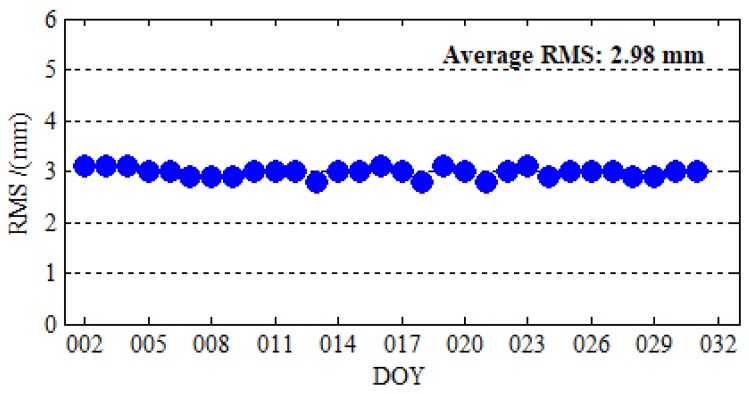
Time series of the RMS between iGMAS ZTD and IGS ZTD products from 2 January 2019 to 31 January 2019.

Figure 10 depicts the distribution and 30-day *RMS* between *iGMAS* and *IGS ZTD* products of 130 *IGS* stations. It is obvious from Figure 10 that the 30-day *RMS* of 130 *IGS* stations are all below 5 mm, and the 30-day *RMS* of most *IGS* stations ranges from 0 to 3.5 mm. From Figure 11, we can find that the stability of the time series of *RMS* and the accuracy of *iGMAS ZTD* products are very good. The average *RMS* of 130 *IGS* stations for 30 days is 2.98 mm. The *IGS ZTD* products are synthesized from the results of several analysis centers, and their accuracy is 1.5 to 5 mm [35]; the difference between *iGMAS* and *IGS ZTD* products are in millimeter level. Therefore, the millimeter accuracies of *iGMAS ZTD* products are sufficient to assess the accuracy of *ZTD* estimated by PPP.

**Figure 12:**
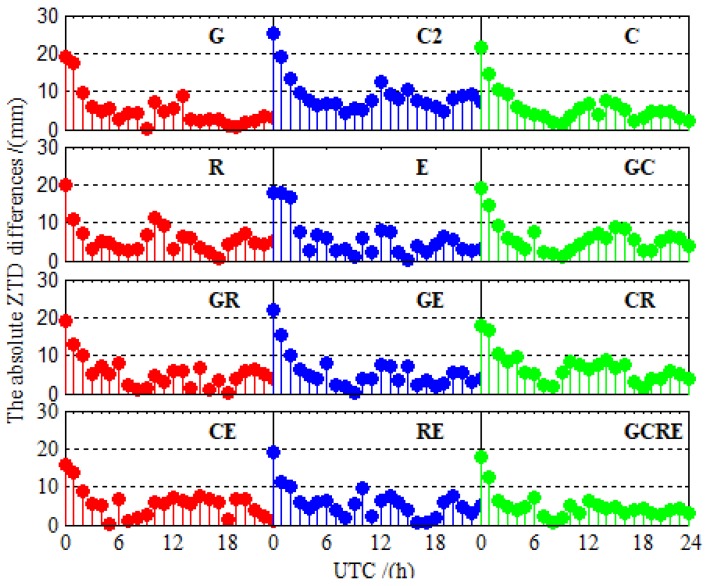
The absolute ZTD differences between ZTD values estimated using PPP and iGMAS ZTD at BRCH for 12 different processing cases on DOY 024 in 2019.

**Figure 13:**
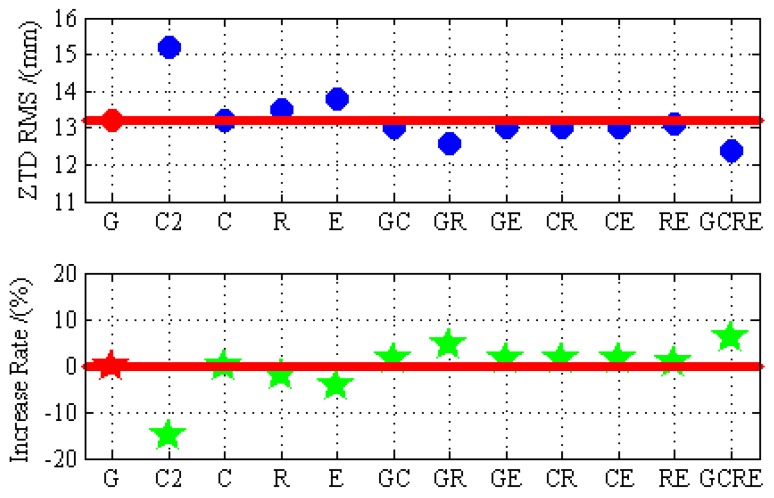
The RMS error of ZTD and the increase rate based on GPS-only results for GPS-only, BDS-2-only, BDS-2/BDS-3, GLONASS-only, Galileo-only, GPS/BDS-2/BDS-3, GPS/GLONASS, GPS/Galileo, BDS-2/BDS-3/GLONASS, BDS-2/BDS-3/Galileo, GLONASS/Galileo, and GPS/BDS-2/BDS-3/GLONASS/Galileo static PPP processing using real observation data collected at 16 stations over 30 days.

In order to assess the accuracy of PPP ZTD, the ZTD products that were provided by iGMAS are used as references to calculate the absolute ZTD differences and RMS between ZTD estimated by PPP and the ZTD products. Figure 12 depicts the absolute ZTD differences between ZTD estimated by PPP and the iGMAS ZTD at BRCH for 12 different processing cases on DOY 024 in 2019. As shown in Figure 12, the absolute ZTD differences preform the maximum at the beginning. ZTD accuracy is as poor as its positioning accuracy when the convergence process is not completed because ZTD is estimated together with the receiver position parameters in an epoch and the technology of PPP needs a certain convergence time [36,37]. The significance of this finding becomes more apparent in Figure 9. With the BDS-3 is open access, the absolute ZTD differences of BDS-2/BDS-3 is smaller than that of BDS-2. More interestingly, the trend of BDS-2/BDS-3 is slightly more stable than that of GLONASS and Galileo. Figure 13 demonstrates this interesting funding well. The ZTD RMS of GLONASS and Galileo are a little worse than that of BDS-2/BDS-3. Now, turn to multi-GNSS, multi-GNSS increases the stability of the solution of ZTD over the single-constellation and dual-constellation PPP. This viewpoint can be confirmed in both Figure 12 (GCRE) and Figure 13.

Figure 13 indicates the RMS statistical values of ZTD and the increase rate that is based on GPS-only results for 12 different processing using real observation data collected at 16 stations over 30 days. As mentioned above, the technology of PPP needs a certain convergence time, the RMS statistical values using the ZTD bias in the second hour to last hour, in which the ZTD solution has reached stable values. We can draw the following points of view based on the results of the selected observation data. The BDS-2/BDS-3 PPP improves the ZTD accuracy by 13.16% over the BDS-2-only PPP. There are no gaps between GPS, GLONASS, Galileo, and BDS-2/BDS-3. Moreover, the GLONASS PPP and Galileo PPP are slightly poorer than the BDS-2/BDS-3 PPP in terms of ZTD accuracy. Additionally, the GPS/BDS-2/BDS-3, GPS/GLONASS, GPS/Galileo, BDS-2/BDS-3/GLONASS, BDS-2/BDS-3/Galileo, GLONASS/Galileo, and GPS/BDS-2/BDS-3/GLONASS/Galileo PPP improves the ZTD accuracy by 1.52%, 4.55%, 1.52%, 1.52%, 1.52%, 0.76%, and 6.06% over GPS-only PPP. The contributions of BDS and Galileo to multi-GNSS are significant.

## 6. Conclusions

As of 1 April 2019, the 33 BDS satellites and 26 Galileo satellites can currently transmit the health signals for users. BDS and Galileo have been transformed into stable and reliable global navigation systems, which can provide global precise services. Therefore, it is meaningful to evaluate the ability of global PPP of the GPS, BDS, GLONASS, Galileo, and multi-GNSS. The performance of the GPS, BDS-2, BDS-2/BDS-3, GLONASS, Galileo, and multi-GNSS were analyzed in terms of PDOP, positioning accuracy, convergence time, and ZTD accuracy. Especially, the contribution of BDS-3 to multi-GNSS was analyzed to identify the performance of BDS. In the geographic area of the selected station, the conclusions are listed, as follows.

The current BDS and Galileo are not regional navigation systems, but global navigation systems. They are basically capable of global precise positioning and the BDS-2/BDS-3 and Galileo PDOP values are fairly evenly distributed around the world, as seem as GPS and GLONASS.

There is no significant difference between GPS, BDS, GLONASS, and Galileo from the perspective of the positioning accuracy and convergence time. The static BDS-2/BDS-3 PPP improves the positioning accuracy by 17.05%, 24.42%, and 35.65% and reduces the convergence time by 27.15%, 27.87%, and 35.76% over the BDS-2-only PPP in three coordinate components, respectively. The RMS of the global position errors for static BDS-2/BDS-3 PPP and Galileo-only PPP are 10.7, 19.5, 20.4 mm and 6.9, 18.6, 19.6 mm, respectively, as the same level as GPS and GLONASS. The GPS/BDS-2/BDS-3 and GPS/Galileo PPP achieves slightly better performance than GPS/GLONASS PPP. Similar to the static PPP, the kinematic BDS-2/BDS-3 PPP improves the position accuracy by 26.32%, 36.00%, and 35.94% and reduces the convergence time by 19.32%, 14.07%, and 19.86% over the BDS-2-only PPP in three coordinate components, respectively. The effect of multi-GNSS to improve the positioning accuracy and the reduce convergence time is more obvious in kinematic positioning. GPS/BDS-2/BDS-3 and GPS/Galileo PPP are both better than GPS-only PPP in terms of kinematic positioning accuracy and convergence time. The multi-GNSS PPP further increases the positioning accuracy and decreases the convergence time over the single-constellation and dual- constellation PPP in both static and kinematic PPP.

The BDS-2/BDS-3 PPP accuracy improves the ZTD by 13.16% over the BDS-2-only PPP and there is no significant difference between GPS, BDS, GLONASS, and Galileo. Moreover, the GLONASS PPP and Galileo PPP are slightly poorer than the BDS-2/BDS-3 PPP. Regarding the multi-GNSS, the GPS/BDS-2/BDS-3, GPS/GLONASS, GPS/Galileo, BDS-2/BDS-3/GLONASS, BDS-2/BDS-3/Galileo, GLONASS/Galileo, and GPS/BDS-2/BDS-3/GLONASS/Galileo PPP improve the ZTD accuracy by 1.52%, 4.55%, 1.52%, 1.52%, 1.52%, 0.76%, and 6.06% over GPS-only PPP. The contributions of BDS and Galileo to multi-GNSS are significant.

In summary, the new generation BDS is basically capable of global PPP. Its coverage and positioning performance are significantly improved when compared with BDS-2, and the difference is significantly reduced as compared with GPS, GLONASS. Galileo has also made good progress in terms of the coverage and precise positioning. BDS and Galileo are basically at the same level as GPS and GLONASS, and they can be well applied to global precise positioning and other application.

## Figures and Tables

**Table 1:** Precise point positioning (PPP) processing strategy.

Items	Models
Observations	Pseudorange and Carrier-phase observations
Signal selection	GPS: L1/L2; BDS-2/BDS-3: B1I/B3I; GLONASS: L1/L2; Galileo: E1/E5a
Sampling rate	30 s
Elevation cutoff	7.5°
Observation weighting	GPS: A priori precision 0.3 m and 0.003 m for code and phase observations, respectively. BDS-2/BDS-3: A priori precision 0.6 m and 0.006 m for code and phase observations, respectively. In view that BDS GEO satellite orbit and clock offset are at a relatively lower accuracy [23,24], their measurements are down-weighted with a factor of ten. GLONASS: A priori precision 0.3 m and 0.0045 m for code and phase observations, respectively. Galileo: A priori precision 0.3 m and 0.006 m for code and phase observations, respectively.
Tropospheric dry delay	GPT3 + VMF3 [25]
Ionospheric delay	Eliminated by IF linear combination
Satellite & Receiver antenna	ANTEX PCO + PCV [26]
Phase windup effect	IERS conventions 2010 [26]
Earth rotation	IERS conventions 2010 [26]
Relativistic effect	IERS conventions 2010 [26]
Station displacement	Solid Earth, Pole and Ocean tide (IERS2010) [26]
Receiver coordinate	Static PPP: Estimated as constant Kinematic PPP: Estimated as white noise process
Receiver clock offset	Estimated as white noise process
Tropospheric wet delay	Estimated as random walk
ISB	Estimated as white noise process
Satellite DCB	BDS: Eliminated by IF linear combination [12] GPS/GLONASS/Galileo: MGEX DCB products [27,28]
Ambiguity	Estimated as constant; float values

**Table 2:** Comparison of GPS, BeiDou navigation satellite system (BDS)-2, BDS-2/BDS-3, GLONASS, Galileo, and multi-Global Navigation Satellite System (GNSS) PPP models.

	**G**	**C2**	**C**	**R**	**E**	**GC**
Observed quantity	2 m	2 o	2 o + 2 n	2 p	2 q	2 m + 2 o + 2 n
parameter	m + 5	o + 5	o + n + 5	p + 5	q + 5	m + o + n + 6
Redundancy	m − 5	o − 5	o + n − 5	P − 5	q − 5	m + o + n − 6
	**GR**	**GE**	**CR**	**CE**	**RE**	**GCRE**
Observed quantity	2 m + 2 p	2 m + 2 q	2 o + 2 n + 2 p	2 o + 2 n + 2 q	2 p + 2 q	2 m + 2 o + 2 n + 2 p + 2 q
parameter	m + p + 6	m + q + 6	o + n + p + 6	o + n + q + 6	p + q + 6	m + o + n + p + q + 8
Redundancy	m + p − 6	m + q − 6	o + n + p − 6	o + n + q − 6	p + q − 6	m + o + n + p + q − 8

**Table 3:** Kinematic PPP accuracy and convergence time.

		**G**	**C2**	**C**	**R**	**E**	**G + C**
Accuracy (cm)	North	4.40	7.98	5.88	4.99	5.46	3.45
	East	5.61	11.61	7.43	6.14	6.06	4.63
	Up	7.12	15.50	9.50	9.51	8.45	6.04
	3 − D	10.08	20.95	13.42	12.37	11.74	8.36
Time (min)	North	56.15	76.80	61.96	58.15	56.57	36.42
	East	65.98	90.43	77.71	67.66	68.51	60.22
	Up	71.24	121.98	97.75	95.57	92.40	58.07
		**G + R**	**G + E**	**C + R**	**C + E**	**R + E**	**G + C + R + E**
Accuracy (cm)	North	3.20	3.24	3.50	3.59	3.45	2.68
	East	4.20	4.37	4.74	5.79	5.13	3.28
	Up	5.61	5.54	5.74	6.18	5.89	5.14
	3 − D	7.70	7.76	8.23	9.20	8.54	6.66
Time (min)	North	33.15	38.11	35.86	40.97	38.91	30.05
	East	45.23	49.70	53.60	46.28	45.91	34.05
	Up	55.91	52.58	66.93	61.57	67.32	41.25
